# Catching's Compendium of Practical Dentistry

**Published:** 1890-08

**Authors:** 


CATCHING'S COMPENDIUM OF PRACTICAL DENTISTRY.—We learn from Dr. Catching that his compilation of practical matter of the current dental literature during the year, is assuming splendid proportions, and is meeting with fine success, being very favorably welcomed by the "wide-awake" dentists. We anticipate a valuable publication from this Compendium and wish it every success. $2.50 per volume. B. H. Catching, D.D.S., Atlanta, Ga. Sold only on subscription.

